# Can one size fit all? Approach to bacterial vaginosis in sub-Saharan Africa

**DOI:** 10.1186/s12941-016-0132-6

**Published:** 2016-03-11

**Authors:** Zenda Woodman

**Affiliations:** Department of Molecular and Cell Biology, University of Cape Town, Rondebosch, Cape Town, South Africa

**Keywords:** Bacterial vaginosis, Sub-Saharan Africa, HIV, Aetiology

## Abstract

Bacterial vaginosis (BV) is the most common vaginal disorder affecting women of reproductive age and is associated with increased risk of sexually transmitted infections such as human immunodeficiency syndrome (HIV-1). Sub-Saharan Africa has the highest BV and HIV-1 burden and yet very few studies have focused on understanding the aetiology of BV and its association with HIV in this region. It has been suggested that we need to accurately diagnose and treat BV to lower the risk of HIV infection globally. However, effective diagnosis requires knowledge of what constitutes a “healthy” cervicovaginal microbiome and current studies indicate that *Lactobacillus crispatus* might not be the only commensal protective against BV: healthy women from different countries and ethnicities harbour alternative commensals. Microbiotas associated with BV have also shown global variation, further complicating effective diagnosis via culture-based assays as some species are difficult to grow. Antibiotics and probiotics have been suggested to be key in controlling BV infection, but the efficacy of this treatment might rely on reconstituting endogenous commensals while targeting a specific species of BV-associated bacteria (BVAB). Alternatively, therapy could inhibit essential BV bacterial growth factors e.g. sialidases or provide anti-microbial compounds e.g. lactic acid associated with a healthy cervicovaginal microbiome. But without global investigation into the mechanism of BV pathogenesis and its association with HIV, selection of such compounds could be limited to Caucasian women from certain regions. To confirm this suggestion and guide future therapy we require standardised diagnostic assays and research methodologies. This review will focus on research papers that describe the global variation of BV aetiology and how this influences the identification of determinants of BV pathogenesis and potential probiotic and antimicrobial therapy.

## Background

Bacterial vaginosis (BV) is associated with sexually transmitted infections (STIs) as well as pelvic inflammatory disease and pregnancy complications [1]. The most alarming association is BV’s relationship with increased risk of HIV infection [2]. The high prevalence of BV in sub-Saharan Africa (approximately 55 % of women) [3, 4] could be a very important contributing factor to the prevalence of HIV infection in this region afflicted with 60 % of global HIV infections (UNAIDS). It has thus been suggested that successful treatment of BV could ultimately lead to lowering HIV infection in this region. Unfortunately, the cause of BV remains unknown although it is generally characterised by the outgrowth of “unhealthy” facultative and obligate anaerobic bacteria with a concomitant decrease in the levels of “healthy” *Lactobacillus* spp. within the genital tract [5]. Given the polymicrobial nature of BV and recent evidence, it is highly likely that pathogenesis of BV-associated bacteria (BVAB) is shaped not only by the bacterial populations present in the genital tract but also by specific host factors. Human genetic host immunity and the identity of “healthy” and “unhealthy” genital tract bacteria differ globally, suggesting that diagnosis and treatment of BV might need to be adjusted according to region. This approach to therapy is unrealistic in resource-poor settings and before we accept this strategy as gold standard we need to confirm our current understanding of “healthy” vaginal microflora and the identity of BVAB. This review aims to highlight the need for studies in sub-Saharan Africa to investigate the aetiology of BV in this region using standardised protocols. Furthermore, understanding the mechanism by which BV increases the risk of HIV will provide new targets for antimicrobial agents.

## What constitutes a healthy cervicovaginal tract microbiome?

Seventy percent of healthy Caucasian females carry predominantly genital *Lactobacilli* spp. [6] with the most common being *L. crispatus*, *L. gasseri*, *L. jensenii* and *L. iners* [7]. Meta-analysis of a number of studies indicated that *L. crispatus* was significantly associated with the absence of BV, and transition to BVAB occurred via outgrowth of *L. iners,* confirming a previous study that *L. gasseri* and/or *L. iners* are associated with BV-related microflora whereas *L. crispatus* protected against dysbiosis [8, 9]. However, studies focused on sub-Saharan countries have indicated that the predominant *Lactobacillus* species varied both within and between countries. Three South African studies reported conflicting results: one indicated that *L. crispatus* was associated with normal cervicovaginal microflora (p = 0.024), supporting studies on Caucasian women, whereas another showed that BV- and HIV-negative women carried predominantly *L. salivarius*. Finally, the last study showed that most women carried both *L. crispatus* and *L. jensenii* and that *L. jensenii* and not *L. crispatus* was associated with lack of BV (p = 0.053) [10–12]. The majority of women from Kenya, Rwanda, South Africa and Tanzania had predominantly genital *L. iners* with coincident anaerobic microbes [13]. Another descriptive cross-sectional study observed no difference between South African and Kenyan women with *L. crispatus* and *L. vaginalis* associated with low Nugent scores [14]. Nigerian women were mostly colonized with genital *L. iners* and *L. gasseri* and Ugandan women carried primarily *L. reuteri*, *L. crispatus*, *L. vaginalis* and *L. jensenii* [15, 16]. Therefore, although *Lactobacilli* were found in women from different countries, the dominant species differed and some healthy women carried non-*Lactobacilli* anaerobic microflora.

These results were confirmed when women from different ethnicities were compared from the same region. It is unknown why black women have a higher prevalence of BV than Caucasians [17, 18]. However, a contributing factor could be that the cervicovaginal microflora of healthy women differs according to race [19]. Srinivasan et al. indicated that 28 taxa were differentially associated with race in the USA (p < 0.05) with *Leptotrichia amnionii*, *Atopobium vaginae* and BVAB1 found in more African-American BV-negative women than Caucasians. Furthermore, the healthy microbiomes of African-American—women were dominated by *L. iners* and those of Caucasians, *L. crispatus* [20]. Healthy microbiomes also varied among Hispanic, African-American, white and Asian women where the cervicovaginal tracts of white and Asian women were dominated by *Lactobacilli* spp. and African-American and Hispanic individuals carried more non-*Lactobacilli* anaerobic bacteria (p < 0.0001) [21]. Interestingly, a study of black South African women also reported that most asymptomatic women carried non-*Lactobacillus* species, similar to African-American women. The dominant *Lactobacillus* spp. was also *L. iners*, suggesting that healthy women of African descent could be less likely to carry cervicovaginal *L. crispatus* [8]. As *L. iners* could play a role in BV pathology, this finding could in part explain the high incidence of BV amongst black women especially in Southern Africa [22]. However, Kenyon et al. cautioned against this suggestion given that the prevalence of BV in some African countries such as Burkina Faso is quite low [23].

BV diagnosis is usually based on four physiological Amsel criteria or Nugent score—a gram stain that determines the relative amounts of gram-positive *Lactobacilli* and gram-negative rods (low score of 0–3 indicating mainly *Lactobacilli*/normal vaginal “flora”; high score of 8–10 indicating BV). Recently, BV-associated dysbiosis was shown not to correlate with three of the four Amsel criteria and the Amsel method was unlikely to identify BV-positive women if they lacked dominant *Lactobacilli* species [13]. This is not reassuring as diagnosis of BV based on Amsel criteria (malodour, discharge, high pH and clue cells) is most commonly used in developing countries.

## How could *Lactobacilli* protect against BV and HIV?

Variation in healthy genital tract *Lactobacilli* commensals across countries and ethnicities could lead to varying levels of protection against BV and HIV-1. Comparative functional genomic studies have shown that *Lactobacillus* spp. have evolved in a species-specific manner to adjust to the cervicovaginal environment, each expressing alternative adaptive factors. Therefore, microbes could influence the health of the genital tract through multiple mechanisms [24] such as the production of bacteriocins, lowering of the genital tract pH, and/or release of hydrogen peroxide [25–27]. *Lactobacilli* produce strain-specific bacteriocins such as reuterin by *L. reuteri* and lactocepin by *L. casei* and *L. paracase* [28]. Despite being the focus of many earlier studies, it is unlikely that hydrogen peroxide plays a role in HIV acquisition as the level of hydrogen peroxide produced by *Lactobacilli i*n the hypoxic environment of the genital tract would be too low to inhibit HIV [29].

Overall, high pH correlated best with high Nugent scores [21] and low pH prevented HIV infection [30]. However, it was shown that lactic acid and not pH was responsible for inhibiting HIV-1 and BVAB [29, 31]. Cervicovaginal microbiome pyrosequencing showed predominance of lactic acid anaerobes in black and Hispanic women, suggesting that the presence of lactic acid could play a very important role in defining healthy vaginas and not a specific bacterial species [21]. Furthermore, the presence of any *Lactobacillus* spp. was associated with lower risk of HIV infection [3] and lower levels of HIV RNA in cervical vaginal lavages (CVLs) [32]. However, another study showed that the level of protection could be strain-specific: *L. crispatus* was better associated with lower HIV RNA levels than *L. iners* [33]. These species rarely co-dominate, probably due to competition and the relative ability of each species to adapt to different environments [24].

Witkin et al. [35] reported that *Lactobacillus* spp. produced either l- or d-lactic acid and only the l-isoform inhibited HIV-1 infection. l-lactic acid induces the IL-23/IL-17 T cell pathway, release of pro-inflammatory cytokines, lymphocyte activation and increase in metalloproteases responsible for disruption of the cervix. The release of different cytokines depended on the species of *Lactobacillus* present [34] and could be due to genetic differences between species as *L. crispatus,L. gasseri* and *L. iners* do not have the same number of copies of the l- and d-lactate dehydrogenase (LDH) genes [35]. Therefore, protection might be *Lactobacillus* spp. dependent and as the commensals of healthy women differ globally, it is likely that microbes other than *Lactobacilli* could be protective via similar mechanisms (production of l-lactic acid) or novel ways, altering the definition of a “healthy” genital tract.

## Could a specific anaerobe predict BV and HIV?

A number of studies have identified different bacteria associated with BV in Caucasian women such as *Veillonella parvula, Bacteroides, Peptococcus asaccharolyticus, Gardnerella vaginalis*, *Mobiluncus* spp., *Mycoplasma hominis* and *Chlamydia trachomatis* [36–38]. Unravelling the BV microbiome using molecular techniques has helped to identify non-culturable bacteria such as *Atopobium vaginae*, newly identified BVAB strains (BVAB1-3), *Megasphaera* spp. and *Leptotrichia* spp. [21]. However, as these bacteria are also lactic acid producers and found in BV-asymptomatic women, it has been suggested that they do not indicate unhealthy vaginas. In sub-Saharan Africa, BV was associated with *G. vaginalis* in Kenya but not in Uganda [39] and *Prevotella bivia* or *Lachnospiraceae* were identified in Tanzania [40]. *Mycoplasma hominis* infected 35 % of HIV-negative Nigerian women whereas *G. vaginalis*, *Prevotella* spp., *Mobiluncus*, *Atopobium* spp. and *E. coli*, which predominate in Caucasians, were not identified [15]. *Mycoplasma* lacks a cell wall and thus cannot be identified using the Nugent scoring system. It is thus possible that this organism is underrepresented in Caucasian BV populations because of the type of diagnostic assay used in some studies [32, 41]. The presence of both *G. vaginalis* and *M. hominis* in the genital tract was associated with increased CVL HIV RNA. When analysis compared these two organisms singly only *Mycoplasma* remained significantly associated with HIV levels (p = 0.0001) [32]. This could suggest that *Mycoplasma* plays an important role in HIV acquisition and that correct screening and diagnostic assays should be used to confirm whether it is associated with BV and HIV globally.

The primary bacteria associated with BV biofilms are *G. vaginalis* and *A. vaginae* [42, 43]. It has been suggested that the genital epithelium is colonised by *G. vaginalis* first and its biofilm production facilitates the colonisation of secondary anaerobes [44–46]. However, as *G. vaginalis* has been isolated from healthy women and introduction of vaginal secretions and not inoculation with pure *G. vaginalis* culture resulted in BV, it was thought to be a component of the normal genital microbiome and thus not the causative agent for BV [47–50]. Machado et al. [45] showed that *G. vaginalis* adherence displaced *L. crispatus*, grew threefold better in the presence of certain anaerobes and encouraged the biofilm growth of mainly *P. bivia*. The authors suggest an interdependent relationship between *Lactobacilli* and BVAB and that this association might be species-specific [46].

Schwebke et al. [20] reviewed convincing evidence as to the role of *G. vaginalis* in BV as nearly 100 % of women with BV carry this specific bacterium whereas other colonising anaerobes are highly heterogeneous. They also suggest that *G. vaginalis* diversity could result in both pathogenic and non-pathogenic strains [45] with only specific biofilm-causing strains responsible for BV [51]. Genomic sequencing and in vitro analysis of two *G. vaginalis* strains- one from a BV-infected woman and the other from a BV-negative women- showed that the former strain was pathogenic with enhanced biofilm production [52]. In support of this theory, Vaginolysin cytotoxicity also varied between *G. vaginalis* strains, reiterating the importance of genetic variation between strains. What this means for BV in sub-Saharan Africa where BV microbiomes differ between countries remains unknown.

## How could BV anaerobes enhance HIV infection?

A heat stable factor found in CVLs of BV-infected women enhanced HIV replication, suggesting that BVAB could increase HIV acquisition directly [53–55]. Vaginolysin produced by *G. vaginalis* facilitates bacterial growth [56] and enhances HIV infection by permeabilising the cervicovaginal epithelium [52].

HIV-1 Envelope (Env) glycosylation may play a role in HIV transmission and thus glycosidases that alter viral glycans could help select specific transmitted founder variants. Dendritic cell DC-SIGN receptor binds to HIV via Env glycans and enables *trans*-infection of CD4+ T cells, thus facilitating HIV transmission [57]. One study showed that CVLs from BV-infected women had higher levels of sialidase, α-galactosidase, β-galactosidase and α-glucosidase than uninfected women, suggesting that BVAB produce enzymes that have the potential to alter the glycome of the genital tract [58]. Using lectin microarray profiling of CVLs of women with and without BV indicated that the number of high mannose N-glycans decreased in the presence of BV [59]. The authors suggest that the high mannose residues on the glycoproteins of the genital mucosa outcompete HIV Env for binding to DC-SIGN or macrophage mannose receptor, preventing infection of macrophages and dendritic cells involved in HIV transmission [60–62].

Bacterial vaginosis was also associated with fewer CVL sialic acid residues as expected with an increase in sialidase levels associated with the onset of BV [63]. Sialidase levels are currently used to diagnose BV using the BVBlue system [64]. Sialidase secreted by *Bacteroides* spp. and *G. vaginalis* help produce biofilms and both mucinases and sialidases are involved in STIs (reviewed by Wiggins) [65] by disrupting the integrity of the mucosa, facilitating the adhesion of pathogens to mucins and/or underlying epithelial cells [66]. The negatively charged sialic acid molecules at the terminal ends of the O-linked sugar chains determine changes in mucosal viscosity [67] influencing viral access to epithelial cells. Sialidases could also directly affect HIV infection as both gp120 and CD4 carry terminal sialic acid residues. In fact, treatment of cells or HIV with sialidase enhanced HIV infection [68–70], suggesting that removal of sialic acids could facilitate virus-target cell binding and thus enhance transmission. Future studies should investigate the significance of this finding on the transmission of HIV by evaluating the effect of sub-Saharan-specific BVAB on CVL sialidase levels of BV-positive women and their impact on HIV replication.

## Could the immune response to BV facilitate HIV transmission?

It has been suggested that the genital tract immune response plays a very important role in the pathophysiological condition of BV [71] because interactions between genital epithelial cells and microbiota regulate the innate immune response. Therefore, disruption of the delicate balance between microbial species could alter pathogen susceptiblity, facilitating HIV replication/shedding in the genital tract and [72] ultimately leading to increased female to male HIV transmission [73, 74]. Schellenberg et al. [75] reviewed studies that indicated that BV-associated inflammation occured via activation of Toll-like receptors (TLRs). Royse et al. [76] indicated that a genetic variation in TLR4, TLR9 and TLR2 of African-American adolescents was associated with recurrence of BV in HIV-infected individuals. A polymorphism in TLR2 was also associated with BV and these authors suggest that specific bacteria could have differential effects on TLRs [77]. Mitchell et al. [34] reviewed findings that showed that different BVAB were associated with varying cytokines and activation of the innate immunity of fully differentiated vaginal epithelial cell aggregates was species-specific: *A. vaginae* increased epithelial cell mucins and pro-inflammatory cytokines; *L. iners* activated pattern-recognition receptor-signaling activity [77] whereas *Prevotella bivia* and *L. crispatus* seemed to have no effect. Therefore infection with *A. vaginae* could induce a pro-inflammatory immune response that disrupts barrier functions whereas other microbes could elicit different responses [78]. This reiterates the need to fully understand genital immunity associated with BV in sub-Saharan Africa, noting the difference in global BV-associated microbiomes discovered thus far.

## Could probiotics and antibiotics treat BV and lower risk of HIV infection?

Treatment of BV with metronidazole did not prevent recurrent BV infections nor lower levels of viral RNA (shed virus) and viral DNA (cell associated) in CVLs [79]. One reason for this is that the biofilm barrier needs to be overcome before anti-microbial agents can gain access to the adherent bacteria. Retrocyclin not only inhibits Vaginolysin and thus prevents biofilm production, it also has anti-HIV activity and is currently being evaluated as an anti-HIV microbicide [80]. To prevent microbicides from altering the genital innate immune response, disrupting the integrity of the mucosal epithelium and enhancing HIV acquisition [81, 82], innate immune regulators and/or antimicrobial agents that eliminate BV-associated microbiota without disrupting beneficial *Lactobacilli* spp. should be included [83]. One potential candidate, carbohydrate binding agents (CBAs) has been shown to prevent CD4 T cell HIV infection, cell–cell fusion, binding to DC-SIGN and *trans*-infection of CD4 T cells without affecting the growth of commensal *Lactobacilli* [84].

Metronidazole and clindamycin do not prevent recurrent BV infections as the *Lactobacilli* population is rarely reconstituted [85]. Probiotics could thus be highly beneficial- modulating the mucosal flora, maintaining the integrity of the epithelial barrier and regulating the immune response. Hydrogen peroxide-producing *Lactobacilli* have been shown to be protective against a number of bacterial infections and have been used in probiotics [86, 87]. Live *Lactobacilli* and the culture supernatant of *Lactobacilli* inhibited HIV infection. The most effective of the *Lactobacilli* tested was *L. gasseri* [88]. As dysbiosis results in inflammation [89], selection of probiotics that do not disturb the natural flora and thus the innate immune response is very important. Numerous studies have tested different *Lactobacillus* strains with varying effects [6]. Homayouni et al. [90] review on probiotic trials between 1990 and 2011 suggested that combination treatment with *L. acidophilus*, *L. rhamnosus*, and *L. fermentum* normalised cervicovaginal microbiome resulting in curing BV and preventing relapse. In contrast, *L. fermentum* and *L. plantarum* were shown by Vicariotto et al. [91] to reduce biofilms in vitro and cure BV in human trials whereas *L. crispatus*, *L. reuteri*, and *L. iners*, disrupted biofilms in another study [92].

Despite this evidence, review and analysis of a number of studies in 2009 indicated that there was insufficient evidence to support the use of probiotics in the treatment of BV and that large randomised trials with standard methodology was still outstanding [93]. Factors that need to be considered are the application of unsuitable bacterial strains and/or colonisation difficulties in the presence of BVAB [86, 94–96]. A randomized double blind study in 2009 indicated that the *L. crispatus* probiotic was able to colonise only in the absence of endogenous *L. crispatus*, lack of condom use and without recent sexual activity, suggesting that the choice of *Lactobacillus* probiotic, the identity of the natural microflora and sexual practices could affect the efficacy of probiotics [96]. Due to the high variability of the genital microbiome in the genital tract of women [35], it is most likely that a single probiotic strain might not be sufficient to prevent BV or HIV infection.

## Conclusion

Studies suggest that BV pathology is highly dependent on *Lactobacilli* spp., BVAB and host genetic differences within the context of social behaviour. This synergy is complicated by differences in *Lactobacilli* spp. and BVAB across race and nationality so that diagnosis and treatment within resource-poor settings such as sub-Saharan Africa requires new consideration. We need to know whether differences across regions and ethnicities reflect true diversity or are due to study design. Thus we need to first standardise a global methodology for BV screening and identification of commensals and BVAB before rigorous longitudinal comparisons between different races and countries are carried out. In conjunction with this, basic research needs to apply in vitro assays that circumvent CVL donor variation to identify novel markers of BV and potential targets for drug design. Without a global approach, controlling BV in sub-Saharan Africa is highly unlikely.
